# Factors affecting food addiction: emotional eating, palatable eating motivations, and BMI

**DOI:** 10.1002/fsn3.4333

**Published:** 2024-07-28

**Authors:** Osman Bozkurt, Ayşe Çamli, Betül Kocaadam‐Bozkurt

**Affiliations:** ^1^ Department of Nutrition and Dietetics, Faculty of Health Sciences Erzurum Technical University Erzurum Turkey

**Keywords:** BMI, emotional eating, food addiction, palatable eating motivations, palatable foods

## Abstract

Evaluating the factors leading to adult food addiction should shed light on potential preventive and treatment strategies for obesity and eating disorders. This research aimed to assess the relationship between food addiction, emotional eating, palatable eating motivations, and the factors that affected them. Five hundred twenty‐two adults participated in this descriptive, cross‐sectional study in Erzurum, Turkey. Participants completed a questionnaire that included a general information form, anthropometric measurements, Palatable Eating Motives Scale (PEMS), Yale Food Addiction Scale (YFAS), and Emotional Eater Questionnaire (EEQ). In total, 181 (34.7%) men and 341 (65.3%) women participated in the study. While 24.7% were overweight or obese, 65.7% had normal BMI (body mass index). Food addiction (FA) was determined in 18.2% of the participants. The FA group had significantly higher PEMS and EEQ scores (*p* < .001). The risk of FA was 3.18 times higher in women than in men (95% CI = 1.65, 6.13, *p* = .001). Significant positive associations between FA, BMI (OR = 1.06, 95% CI = 1.00, 1.11, *p* = .021), and EEQ (OR = 1.31, 95% CI = 1.23, 1.38, *p* = .000) were found. Emotional eating behavior and palatable eating motivations are more common in individuals with food addiction than nonfood addiction. Female gender, emotional eating, and high BMI values were determined as risk factors for food addiction.

## Graphical abstract

Emotional eating behavior and palatable eating motivations are more common in individuals with food addiction than nonfood addiction. Female gender, emotional eating, and high body mass index (BMI) values were determined as risk factors for food addiction.

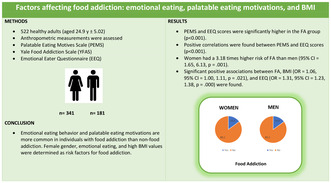

## 1 INTRODUCTION

Globally, obesity is an important health issue. The prevalence of obesity has almost tripled worldwide since 1975. Adult obesity rates in 2016 were 13% and overweight rates were 39% (NCD Risk Factor Collaboration, 2016). According to the World Health Organization (WHO) European Regional Obesity Report 2022, Turkiye has the highest obesity prevalence (32.1%) among European nations (WHO, 2022).

The sustainability of weight loss with modern treatments remains challenging (van Baak & Mariman, 2019). Achievement of weight loss and maintenance depends on an individual's ability to change eating habits (Kheniser et al., 2021). Diverse dietary patterns are related to weight gain, such as overeating and eating without feeling hungry (Bouhlal et al., 2017). Food consumption is the primary factor that can satisfy the body's energy needs, but for many people, eating has evolved beyond simply satisfying their metabolic demands (Şarahman Kahraman & Akcil Ok, 2022; Timper & Brüning, 2017). Various terms that have become widely used in the literature, like “emotional eating,” “stress‐induced eating,” “hedonic eating,” and “food addiction,” are indicative of this. Many people perceive nutrition as a means of connecting with others, occasionally as a way to deal with a challenging circumstance or mood, and occasionally as a rewarding activity (Bilici et al., 2020). Foods processed and made palatable by adding a lot of fat, sugar, or salt are commonly consumed under these circumstances (Gearhardt & Schulte, 2021). Therefore, these foods promote overeating, which is the leading reason behind obesity and associated metabolic diseases compared to other foods (Bjorlie et al., 2022).

Emotional eating behavior has been defined as a psychological eating problem that typically results in overeating in reaction to mood swings like loneliness, depression, and anxiety (Ambwani et al., 2015). Foods defined as palatable create a consumption desire similar to addictive substances like alcohol and cocaine, as they have the potential to disrupt the limbic system's reward mechanism (Wieland, 2019). Consistently high consumption of palatable foods can lead to food addiction, resulting in uncontrolled eating habits (Lindgren et al., 2018). Long‐term consumption of delicious foods can increase the desire to reach these foods later and experience deprivation syndromes in the absence of nutrients (Lennerz & Lennerz, 2018).

Because not all obese individuals consume palatable foods for the same reasons, it may be possible to develop more effective treatments by determining a person's specific motives for eating these foods (Boggiano et al., 2014). Individuals are only occasionally aware of the motivations underlying their actions; being aware of them is frequently the first step in changing eating behavior (Björkman et al., 2022). The Palatable Eating Motives Scale (PEMS) was developed to understand why people eat palatable foods. It also focuses solely on the consumption of palatable food, not the consumption of whole foods in general (Burgess et al., 2014). PEMS is a questionnaire that raises awareness of the person being tested and the person doing the test about the motivations behind the person's consumption of highly palatable foods or beverages (And et al., 2018). It has been reported that in individuals who engage in emotional eating behavior, coping motives (the inclination to consume palatable foods in order to deal with difficulties, concerns, or emotions) are associated with higher body mass index (BMI) (Boggiano, 2016; Boggiano et al., 2015, 2017; Burgess et al., 2014). In addition, it has been stated that the motivation to cope is high in people with food addiction (Schulte & Gearhardt, 2021). Therefore, evaluating the factors leading to adult food addiction should shed light on potential preventive and treatment strategies for obesity and eating disorders. This study evaluated the relationship between food addiction, emotional eating, palatable eating motivations, and the factors that affected them in a Turkish population.

## 2 MATERIALS AND METHODS

### 2.1 Study design and participants

This cross‐sectional study was carried out with 522 adults (19–64 years old) who consented to participate and were selected by the snowball sampling method in Erzurum/Turkiye. Data for the study were collected via an online questionnaire. Individuals with any health problem were excluded from the study. “Ethics Committee Approval” was obtained from Erzurum Technical University Ethics Committee (Meeting Number: 11, Decision Number: 01, 2023) in order to conduct the research. The research was conducted in compliance with the principles outlined in the Declaration of Helsinki. All participants provided informed consent.

### 2.2 Collection of data

Various measures were used for evaluating the relationship between food addiction, emotional eating, palatable eating motivations, and the variables that influenced them, such as age, gender, education duration, current smoking status, and BMI. In this regard, the questionnaire form included a general information form, anthropometric measurements, the Palatable Eating Motives Scale, the Yale Eating Addiction Scale, and the Emotional Eater Questionnaire (EEQ).

### 2.3 General information and anthropometric measurements

The general information form included questions evaluating sociodemographic characteristics (age [year], gender [men/women], education duration [year]), health problems (yes/no), and currently smoking status (yes/no). The participants self‐reported their height (cm) and body weight (kg). The body mass index (BMI) was determined by dividing the body weight by the square of the height, and evaluated using the World Health Organization's BMI classification (WHO, 2000).

### 2.4 Palatable Eating Motives Scale

The Palatable Eating Motives Scale (PEMS) was developed by Burgess et al. (2014) to assess motivations for eating various tasty foods and beverages and revised by Boggiano (2016). And et al. (2018) carried out the scale's Turkish adaptation, validity, and reliability (And et al., 2018). The scale has four subscales and 20 items: Social (pertaining to consuming delicious food or drinks in social situations), Coping (related to consuming tasty items to deal with negative emotions), Reward enhancement (related to consuming delicious food or drinks to amplify positive feelings or experiences, or because of their innately rewarding qualities that have nothing to do with social circumstances), and Conformity (connected to eating delicious food due to external pressure to do so). On a Likert scale, the questions are answered (“1” Almost never/Never, “2” Some of the time, “3” Half of the time, “4” Most of the time, and “5” Almost always/Always). The scale does not have a reverse item. All items are scored between 1 and 5, and the sum of each item was calculated to give the overall mean for each subscale. Cronbach's alpha values of Social, Coping, Reward enhancement, and Conformity subscales were determined as 0.81, 0.90, 0.86, and 0.86, respectively (And et al., 2018).

### 2.5 Yale Food Addiction Scale

The Yale Food Addiction Scale determines individuals' addiction to high‐fat and sugary foods. It was developed by Gearhardt et al. (2009). The scale's Turkish validity, reliability, and adaptation were carried out by Buyuktuncer et al. (2019) (Cronbach's alpha 0.464–0.820) and Bayraktar et al. (2012) (Cronbach's alpha 0.93). The diagnostic scale for food addiction consists of items pertaining to seven symptoms and impairment criteria. Also, the questionnaire asks about the foods that people crave the most. The items scored use a dichotomous and Likert scale and measure addictive‐like eating behaviors toward certain types of foods in the last 12 months, using the DSM‐IV (Diagnostic and Statistical Manual of Mental Disorders, fourth edition) diagnostic criteria. When at least three of the seven symptoms and the impairment or distress criteria are satisfied, a diagnosis of food addiction (FA) can be made.

### 2.6 Emotional Eater Questionnaire

The Emotional Eater Questionnaire (EEQ) was developed by Garaulet et al. (2012) to assess emotional eating behaviors (Garaulet et al., 2012). The Turkish adaptation, validity, and reliability of the scale were carried out by Arslantaş et al. (2020). The scale has three subscales and 10 items: Disinhibition (e.g., How often do you think that food dominates your life instead of the other way around?), Type of food (e.g., Do you find it hard to give up sweets, especially chocolate?), and Guilt (e.g., When you consume “forbidden” foods, such as snacks or sweets, do you feel guilty?). Response options for the questions are measured on a Likert scale, ranging from (“0” Never, “1” Sometimes, “2” Usually, and “3” Always). The scale does not have a reverse item. Increased scores on the subscale suggest higher emotional eating behavior. The Cronbach's alpha coefficient value of the scale was 0.84. Cronbach's alpha values of the subscales of Disinhibition, Type of food, and Guilt were determined as 0.81, 0.57, and 0.64, respectively (Arslantaş et al., 2020).

### 2.7 Statistical analysis

Data analysis was performed using SPSS version 22.0 software (IBM Corpn., Armonk, NY, USA). The variables were assessed to determine whether or not they were normally distributed using visual analysis (histogram and probability graphs), skewness and kurtosis, and Kolmogorov–Smirnov tests. For continuous variables, the mean (X¯) and standard deviation (SD) were utilized, while frequency (*n*) and percentage (%) were used to represent categorical variables. The Chi‐square test, the Student's *t*‐test, or the Mann–Whitney *U* test was used to compare the data. The correlation between the parameters was analyzed using the Spearman or Pearson correlation coefficient, based on the distribution of the data. A binary logistic regression analysis was conducted to evaluate the effect of risk factors on the presence or absence of food addiction and calculate the odds ratio (OR) and 95% confidence interval (CI). FA (code: 0—No FA; 1—FA) was used as the dependent variable, gender (code: 0—men, 1—women), BMI (kg/m^2^), smoking status (code: 0—yes, 1—no), and EEQ total score were used as independent variables. Using the Hosmer–Lemeshow test, the −2 log‐likelihood test, and the Nagelkerke *R* ^2^ test, the logistic regression model was assessed (Hosmer & Lemeshow, 2000; Nagelkerke, 1991). The statistical significance level was set at *p* < .05.

## 3 RESULTS

The mean age of the individuals was 24.9 ± 5.02 years, where 181 (34.7%) were men and 341 (65.3%) were women. The mean education period of the participants was 15.8 ± 1.27 years. While 24.7% were overweight or obese, 65.7% had normal body weight. Participants (24.1%) declared that they smoked currently. The mean scores of the participants in the Social, Coping, Reward enhancement, and Conformity subscales for the PEMS were determined as 2.3 ± 0.99, 2.2 ± 0.90, 2.4 ± 1.04, and 1.5 ± 0.60, respectively. The EEQ total score and the scores for Disinhibition, Type of food, and Guilt subscales were determined as 10.7 ± 2.1, 6.2 ± 2.1, 2.9 ± 1.4, and 1.7 ± 0.49, respectively (Table 1).

**TABLE 1:** General characteristics of the participants (*n*: 522).

	*n*	%
Gender		
Men	181	34.7
Women	341	65.3
Smoking status		
Yes	126	24.1
No	396	75.9
BMI classification		
Underweight	50	9.6
Normal	343	65.7
Overweight	89	17.0
Obese	40	7.7
	**X¯ ± SD**	
Age (year)	24.9 ± 5.02	
Education duration (year)	15.8 ± 1.27	
PEMS		
Social	2.3 ± 0.99	
Coping	2.2 ± 0.90	
Reward enhancement	2.4 ± 1.04	
Conformity	1.5 ± 0.60	
EEQ total score	10.7 ± 2.1	
Disinhibition	6.2 ± 2.1	
Type of food	2.9 ± 1.4	
Guilt	1.7 ± 0.49	

Abbreviations: BMI, Body mass index; EEQ, Emotional Eater Questionnaire; PEMS, Palatable Eating Motives Scale.

Food addiction (FA) was determined in 18.2% of the participants. The proportions were 14.9% for men and 19.9% for women. The age and education period of the participants did not differ according to the FA groups (*p* > .05). PEMS, EEQ, and subscales were significantly higher in the FA group (*p* < .001) (Table 2). While the EEQ total score was 17.5 ± 5.28 in the group with food addiction, it was 9.2 ± 3.96 in the group without food addiction (*p* < .001). The rate of craving for any food in the sample was 85.2%. The three most craved foods in the FA group were chocolate/wafers (58.1%), chips (49.2), and cake (38.9%). Significant positive correlations were found between PEMS and EEQ scores (*p* < .001) (Table 3).

**TABLE 2:** Evaluation of variables according to the food addiction status.

	Food addiction		
	Nonaddiction, *n* (%)	Addiction, *n* (%)	*p*
Gender			
Men	154 (85.1%)	27 (14.9%)	.001^a^
Women	273 (80.1%)	68 (19.9%)	
Smoking status			
Yes	89 (70.6%)	37 (29.4%)	<.001^a^
No	338 (85.4%)	58 (14.6%)	
BMI classification			
Underweight	48 (96.0%)	2 (4.0%)	<.001^a^
Normal	296 (86.3%)	47 (13.7%)	
Overweight	66 (74.2%)	23 (25.8%)	
Obese	17 (42.5%)	23 (57.5)	
	**X¯ ± SD**	**X¯ ± SD**	
Age (year)	24.1 ± 5.26	25.7 ± 3.69	.159^b^
Education duration (year)	15.9 ± 0.90	15.5 ± 2.29	.075^b^
PEMS			
Social	2.2 ± 0.93	2.9 ± 1.07	<.001^b^
Coping	2.1 ± 0.89	3.0 ± 1.06	<.001^b^
Reward enhancement	2.3 ± 0.98	2.9 ± 1.13	<.001^b^
Conformity	1.3 ± 0.48	2.0 ± 0.75	<.001^b^
EEQ total score	9.2 ± 3.96	17.5 ± 5.28	<.001^b^
Disinhibition	5.2 ± 2.28	10.5 ± 4.43	<.001^b^
Type of food	2.6 ± 1.33	4.1 ± 1.35	<.001^b^
Guilt	1.5 ± 1.28	2.9 ± 1.76	<.001^b^

^a^ Chi‐square test.

^b^ Student's *t*‐test, or Mann–Whitney *U* test.

**TABLE 3:** Evaluation of the relationship between PEMS and EEQ (correlation coefficient = *r*).

	EEQ	Disinhibition	Type of food	Guilt
PEMS				
Social	.353**	.345**	.359**	.155**
Coping	.584**	.585**	.495**	.316**
Reward enhancement	.427**	.418**	.466**	.157**
Conformity	.369**	.348**	.268**	.301**

Abbreviations: EEQ, Emotional Eater Questionnaire; PEMS, Palatable Eating Motives Scale; *r*, Spearman or Pearson correlation coefficient.

** *p* < .001.

The value of the regression model −2 log‐likelihood test with predictors was *x* ^2^: 173.302, <.001; the model was described at 46.1% (Nagelkerke *R* ^2^ = .461). The Hosmer–Lemeshow goodness‐of‐fit test had a significant probability greater than 0.05 (*x* ^2^: 3.054, *p* = .931), so the regression model was considered significant. Women had a 3.18 times higher risk of food addiction than men (95% CI = 1.65, 6.13, *p* = .001). Significant positive associations between FA, BMI (OR = 1.06, 95% CI = 1.00, 1.11, *p* = .021), and EEQ (OR = 1.31, 95% CI = 1.23, 1.38, *p* = .000) were found (Table 4).

**TABLE 4:** Evaluation of risk factors for food addiction by binary logistic regression analysis.

	Food addiction				
	*β*	SE	*p*	OR	95% CI
Constant	−6.734	0.817	.000	0.001	
Gender	1.158	0.335	.001	3.18	1.65–6.13
BMI (kg/m^2^)	0.059	0.026	.021	1.06	1.00–1.11
Smoking status	−0.311	0.332	.349	0.73	0.38–1.40
EEQ total score	0.271	0.029	.000	1.31	1.23–1.38
−2LL (*x* ^2^, *p*)	321.973 (173.302, <.001)				
Nagelkerke *R* ^2^	.461				

*Note*: FA (code: 0—No FA; 1—FA); Gender: 0—men, 1—women; Smoking status: 0—yes, 1—no.

Abbreviations: −2LL, −2 Log‐Likelihood; BMI, Body mass index; CI, Confidence interval; EEQ, Emotional Eater Questionnaire; OR, Odds ratio; PEMS, Palatable Eating Motives Scale; SE, Standard error.

## 4 DISCUSSION

Our results study showed female gender, high BMI values, and emotional eater scores as risk factors for food addiction. Furthermore, positive correlations were found between PEMS and EEQ scores.

Palatable foods can be consumed for many reasons (Pietrabissa et al., 2024). The PEMS evaluates the tendency of individuals to consume palatable foods for social reasons, to deal with negative mood, or to reward themselves for improving their mood, or because of external pressures (Pietrabissa et al., 2024). A study on 2434 adult individuals showed a positive correlation between PEMS score and emotional appetite (Bilici et al., 2020). Coping motives were the main focus of the majority of recent studies on self‐reported eating motives and emotion regulation. The consumption of foods high in sugar and fat increased in response to stress (Ambwani et al., 2015). Also, people with less developed emotional regulation abilities might eat more palatable foods in an effort to feel more rewarded (Medina et al., 2023). As expected in this study, positive correlations between PEMS subscales and EEQ scale total and subscale scores were found. These results confirm that individuals may consume palatable foods to manage their emotional states.

Studies have shown that the consumption of palatable foods is associated with food addiction and emotional eating. It has been shown that people with food addiction demonstrated higher palatable food consumption (Schulte & Gearhardt, 2021; Varnado et al., 2024). According to a study conducted by Taş and Gezer (2022) with 275 university students aged 19–28 years, a moderately positive relationship was determined between PEMS and Yale Eating Addiction Scale scores (Taş & Gezer, 2022). Individuals who experience addiction‐like eating have been reported to consume excessively processed foods, especially to deal with negative emotions (Joyner et al., 2015; Schulte & Gearhardt, 2021). In our study, the PEMS subscale scores of people with food addiction were higher than those of people without food addiction. Also, individuals with food addiction received the highest score from the PEMS coping subscale. Additionally, studies have demonstrated a relation between emotional eating and food addiction (Akdeniz et al., 2024; Borisenkov et al., 2020). It is stated that the increase in EEQ scores indicates that the effect of emotional states on food intake is increased, especially for foods that may be considered forbidden, like foods high in fat and sugar (Arslantaş et al., 2020). Our findings indicated that a higher EEQ score was associated with a higher risk of food addiction.

In a study, young adults classified as food addicts consumed higher amounts of ultra‐processed foods and lower amounts of unprocessed foods than nonaddicts (Whatnall et al., 2022). In another study, the consumption of foods like pizza/lahmacun/doner, pastry/bread, french fries, cake, and candy was higher in the group with food addiction (Mengi Çelik & Karaçil Ermumcu, 2022). Similarly, foods high in fat and sugar, such as chocolate, cake, chips, and pizza, were determined to be the most addictive foods in the general population (Mutlu & Mutlu, 2022). Consistent with these studies, it was determined that the most desired foods in people with food addiction were chocolate/wafer (58.1%), chips (49.2%), and cake (38.9%). An intense desire to consume fatty and high‐energy foods, defined as palatable foods and continued high consumption, alters the nucleus accumbens (NAc) function, located in the rostral and ventral forebrain (Ferrario, 2017; Taş & Gezer, 2022). Since the NAc is the main site mediating reward behavior, it is thought to be directly involved in rewarding and addictive behaviors in response to substance use (Ferrario, 2017). Foods defined as palatable create a consumption desire similar to addictive substances such as alcohol and cocaine, due to their potential to disrupt the reward pathway in the limbic system (Wieland, 2019). Therefore, high consumption of palatable foods can lead to food addiction, resulting in uncontrolled eating habits.

In a recent study in Sakarya/Turkiye, the prevalence of food addiction was 14.9% (Akdeniz et al., 2024). In a meta‐analysis study, FA prevalence in children and adolescents was 15% (95% CI 11–19%) for all samples and 19% (95% CI 14–26%) for overweight/obese samples (Yekaninejad et al., 2021). Food addiction was determined in 18.2% of the participants in our study. Although food addiction and obesity are not synonymous, the higher prevalence of food addiction in this population has given rise to the theory that food addiction is a behavioral phenotype that is clinically significant in obesity (Davis, 2017). In many human studies, it has been found that the rates of food addiction are higher in overweight and obese individuals than in individuals with normal weight (Gearhardt & Hebebrand, 2021; Hebebrand & Gearhardt, 2021; Mutlu & Mutlu, 2022). According to a preclinical study, prior to any dietary intervention, rats exhibiting the strongest motivational reactions to a food stimulus gain the greatest increase in body weight when provided with unrestricted consumption of a high‐fat, high‐sugar, “junk food” diet (Robinson et al., 2015). Also, it was demonstrated that rats with a tendency to become obese were more susceptible to the stimulating effects of cocaine, suggesting an enhanced sensitivity of the brain's reward system, and exhibited higher levels of activity in specific neurons in the nucleus accumbens core (NAc) before onset of obesity (Oginsky et al., 2016). In this regard, increases in NAc responsivity may increase individuals' sensitivity to the motivational properties of food cues. Our data show that higher BMI is associated with a higher risk of food addiction; moreover, food addiction may also be a risk factor for obesity.

Female gender is also considered as another risk factor for food addiction. Although some studies (Grammatikopoulou et al., 2018; Wiedemann et al., 2018) reported that there was no significant relationship between food addiction and gender, in more studies, the rates of food addiction were found to be significantly higher in women than in men (Borisenkov et al., 2020; Mengi Çelik & Karaçil Ermumcu, 2022; Taş & Gezer, 2022). Similarly, our study determined that the risk of food addiction in women was 3.18 times higher than in men. Gender‐specific variations in hormonal profiles and/or dietary habits could be the cause of this result (Pursey et al., 2014). In order to evaluate the effect of gender on food addiction, studies that evaluate nutritional status, dietary intake, and biochemical parameters are recommended.

It is stated that individuals with food addiction exhibit an addiction to highly processed foods similar to substance addiction (Gearhardt & DiFeliceantonio, 2023). Smoking and food addiction have common addiction mechanisms. Both use the rewarding pathway of dopamine. The ventral striatum of the basal ganglia in the forebrain and the ventral tegmental area in the midbrain are connected by the mesolimbic dopamine pathway. Smoking and overeating palatable foods increase dopaminergic transmission to the NAc, resulting in positive effects via the brain's reward system; as a result, a sense of pleasure is felt. Dopamine receptors are downregulated over time, and rather than pleasure and homeostatic needs, the need to prevent withdrawal symptoms drives food intake. Cravings and withdrawal symptoms occur if no additional intake is made (Meule, 2011; Wieland, 2019). The results of studies on the relationship between food addiction and smoking in the literature are contradictory. In a study conducted with 630 participants in which the prevalence of food addiction in obese and nonobese individuals was investigated, the rate of food addiction was found to be higher in smokers (Mutlu & Mutlu, 2022). However, Lemeshow et al. (2018) showed that nurses who smoked were less likely to be diagnosed with food addiction. The fact that food and cigarettes can compete with each other for the same neurotransmitters in the brain, such as dopamine, helped to explain this result (Lemeshow et al., 2018). In this study, smoking status was not a risk factor for food addiction. Furthermore, the prevalence of FA was found to be lower in smokers. This result may be found because the smoking frequency was not questioned in detail. The effect of smoking frequency on food addiction should be evaluated in future studies.

The study has the following strengths: large sample size; it is one of the first studies in which the relationship between food addiction, emotional eating, and palatable eating motivations is indicated, and the factors (age, gender, education duration, smoking status, and BMI) that affected them are also clarified; and it is a study that determined that female gender, emotional eating, and high BMI values are risk factors for food addiction. When evaluating the study's data, some limitations should be addressed. First of all, the study was cross‐sectional in nature, meaning that while it was unable to determine a cause‐and‐effect relationship, it was still utilized to assess how the measured variables related to one another. Second, self‐reporting was used to collect the data, which raises the possibility of recall bias and false reporting (Fadnes et al., 2009). Third, measurements of the participant's height and body weight were recorded based on the self‐reports. Finally, this study sample included adults living in Erzurum/Turkey, so these results cannot be generalized to all adults worldwide. As a result, it is important to repeat the research in other regions/countries.

## 5 CONCLUSIONS

This study showed that emotional eating behaviors and palatable eating motivations were more common in individuals with food addiction. In addition, the risk of food addiction increased as emotional eater scores and BMI increased. Another significant result of the study was that the risk of food addiction was higher in women. There was a positive relationship between emotional eating and palatable eating motivations. Determining the motivation of individuals with food addiction to eating palatable foods and evaluating them in terms of emotional eating may enable clinicians or researchers to set more specific targets for cognitive–behavioral treatments.

## AUTHOR CONTRIBUTIONS

**Osman Bozkurt:** Conceptualization (equal); data curation (equal); formal analysis (lead); investigation (lead); writing – original draft (lead); writing – review and editing (lead). **Ayşe Çamli:** Data curation (equal); visualization (lead); writing – original draft (equal); writing – review and editing (equal). **Betül Kocaadam‐Bozkurt:** Conceptualization (equal); data curation (supporting); writing – original draft (supporting); writing – review and editing (supporting).

## FUNDING INFORMATION

None.

## CONFLICT OF INTEREST STATEMENT

The authors have declared no conflicts of interest.

## ETHICAL APPROVAL

“Ethics Committee Approval” was obtained from Erzurum Technical University Ethics Committee (Meeting Number: 11, Decision Number: 01, 2023) in order to conduct the research. The research was conducted in compliance with the principles outlined in the Declaration of Helsinki. All participants provided informed consent.

## Data Availability

The datasets generated and/or analyzed throughout this study are publicly unavailable.
